# Future Health Risk Assessment of Exposure to PM_2.5_ in Different Age Groups of Children in Northern Thailand

**DOI:** 10.3390/toxics11030291

**Published:** 2023-03-22

**Authors:** Teerachai Amnuaylojaroen, Nichapa Parasin

**Affiliations:** 1 Department of Environmental Science, School of Energy and Environment, University of Phayao, Phayao 56000, Thailand; teerachai.am@up.ac.th; 2 Atmospheric Pollution and Climate Research Unit, School of Energy and Environment, University of Phayao, Phayao 56000, Thailand; 3 School of Allied Health Science, University of Phayao, Phayao 56000, Thailand

**Keywords:** children health, health risk assessment, exposure, PM_2.5_, infants

## Abstract

Particulate matter with a diameter less than 2.5 (PM_2.5_) is one of the major threats posed by air pollution to human health. It penetrates the respiratory system, particularly the lungs. In northern Thailand, the PM_2.5_ concentrations have significantly increased in the past decade, becoming a major concern for the health of children. This study aimed to assess the health risk of PM_2.5_ in different age groups of children in northern Thailand between 2020 and 2029. Based on the PM_2.5_ data from the simulation of the Nested Regional Climate Model with Chemistry (NRCM-Chem), the hazard quotient (HQ) was used to estimate the possible risk from PM_2.5_ exposure in children. In general, all age groups of children in northern Thailand will tend to experience the threat of PM_2.5_ in the future. In the context of age-related development periods, infants are at a higher risk than other groups (toddlers, young children, school age and adolescents), but adolescents also have a lower risk of exposure to PM_2.5_, albeit maintaining a high HQ value (>1). Moreover, the analysis of risk assessment in different age groups of children revealed that PM_2.5_ exposure might indeed affect adolescent risk differently depending on gender, with males generally at a heightened risk than females in adolescence.

## 1. Introduction

PM_2.5_ is a significant air contaminant that has a significant impact on human health [1]. Due to their small sizes and large interfacial areas, PM_2.5_ particles can be transported by a wide variety of toxic substances, passing through the nose’s purification process, attaining the tip of the nasal passages with the movement of air, and acquiring through spread, permanent havoc toward other parts of the human body via the respiratory system’s air exchange [2]. Fine particulate exposure could potentially impair pulmonary function and aggravate asthma and cardiovascular disease [3]. According to recent reports of global premature mortality rates, Southeast Asia (SEA) accounts for around 25% of global deaths [4]. Asia is a region that has gained notoriety for its stated susceptibility to a greater mortality of about more than 59% of the total global deaths due to poor air quality [5,6,7]. In recent years, significant haze outbreaks in Southeast Asia (SEA) have become increasingly common and severe due to particle pollution. Periodic biomass burning and airborne contaminants from anthropogenic activities greatly pollute the atmosphere in Southeast Asia (SEA) and northern Thailand [8,9].

Air pollution has a severe impact on the health of children. Air pollution contributes to unfavorable preterm birth, infant mortality, impaired respiratory symptoms, allergies, delay gross motor development, cancers, and raising the chance of neurological diseases [10,11]. Because their brains, lungs, and other systems are still growing, children are biologically more susceptible to air pollution than adults [11]. In addition, children are physiologically more vulnerable to polluted air than adults because they inhale twice as fast and frequently through their mouths, absorbing more toxins [11]. Recent research has found that both sudden and long-term exposure to particulate matter increases the risk of death and mortality, with a significant positive relation between PM_2.5_ and child mortality [12]. For instance, Lien et al. [12] demonstrated a positive correlation between infants and PM_2.5_ and premature death in 45 Asian and African nations. Gouveia et al. [13], who assessed the influence of air pollution, found that their outcome indicates a correlation between air pollution and childhood mortality in Latin America. Sarkodie et al. [14] investigated the association between PM_2.5_, mortality, and life expectancy in Europe and North America. Their results demonstrated a statistically strong positive correlation between air pollution, death, and living standards in the nations under consideration. Furthermore, health impacts vary based on the metal’s oxidation state (i.e., copper (Cu), iron (Fe), manganese (Mn), and zinc (Zn)0, altering the absorption, membrane transport, excretion, and toxicity at the cellular or molecular target [15,16]. Children are a particularly vulnerable population to PM_2.5_ exposure and associated metal components. Children inhale more pollutants per unit body weight than adults, and their lungs are still developing, therefore pollutants ingested can interfere with normal lung function development [17]. Additionally, children have narrower airways than adults; while PM_2.5_ may cause moderate irritation in adult airways, it can create significant blockages in children [18]. Children have substantially higher rates of heavy metal absorption and hemoglobin sensitivity to these metals than adults. Studies on Asian countries that have investigated the connection between PM_2.5_ and childhood mortality in children under the age of five found the highest incidence of death as a result of biomass and dust mixture in PM_2.5_ [12]. This is despite the fact that Asian countries experience disastrous air pollution and its influence on mortality rates in children. Studies have classified PM_2.5_ as the most harmful fine particulates in relation to child death [19,20], noting that newborns (0–1 year) and children between the ages of five became more vulnerable to poor air quality [21]. Children are more affected by polluted air than adults because they spend more time outdoors and breathe through their mouths, which increases their exposure [13].

This study evaluated the children’s exposure to PM_2.5_ in northern Thailand from 2020 to 2029. This is because a prior study conducted by Amnuaylojaroen et al. [1] to analyze the health risks related to PM_2.5_ in northern Thailand over 2020–2029 indicated that children tend to be at a greater risk than adults. However, the outcomes in the context of children were limited. In the study, no specific age range of children was considered because specific age groups of children are differently exposed to ambient toxins via breathing. Infants and children, for instance, have a faster metabolism at rest and a greater amount of oxygen uptake per unit body weight than adolescents because of their larger cooling surface per unit body weight and rapid growth [11]. Therefore, the effects of either indoor or outdoor air pollution might differ depending on the age and mobility of a child. To successfully protect and promote better health in children, risk assessments of air contaminants in specific age groups of children should be considered when planning and improving air quality policies. The exposure of pollutants to human can occur through different pathways, which include ingestion, dermal, and inhalation [22]. The exposure of particulate matter (PM) has been recognized as a well-known human health risk factor [22]. In this study, we addressed the non-carcinogenic risk factors of future PM_2.5_ via the inhalation exposure of different age groups in children evaluated in northern Thailand between 2020 and 2029. As a result, the findings of this study will provide health care professionals and the general public with a better understanding of air quality, promoting awareness about the toxicity of PM_2.5_ in children.

## 2. Materials and Methods

To study the health risks of PM_2.5_ exposure in different age groups of children in northern Thailand during 2020–2029, we calculated the HQ value using the PM_2.5_ input data from the previous simulation [22] by the NRCM-Model to quantify the risks resulting from PM_2.5_ exposure in northern Thailand.

### 2.1. Description of Study Area

**Figure 1:**
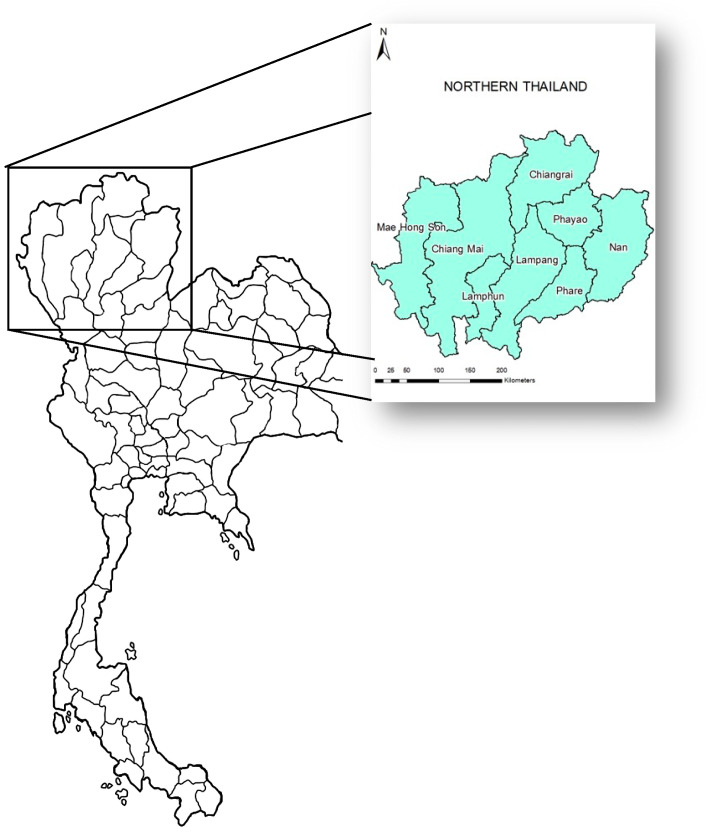
Map of Thailand demonstrating the study area in northern Thailand.

Figure 1 shows the study area in northern Thailand that is generally located in the northern peninsula of SEA and is geographically defined by many mountain ranges bordering Laos and Burma. This region has traditionally experienced air pollution driven by the burning of agriculture waste and forest fires, which release many sizes of particles over the entire year. The primary sources of air pollution in northern Thailand come from both transboundary and domestic burning [8]. Most of the air pollution in northern Thailand is caused by the burning of forests, which primarily occur in deciduous forests. Several regions in northern Thailand were forced to close in 2023 because of haze pollution. According to the Ministry of Public Health, 376,165 people were affected by air pollution-related health concerns, a rise of 163,491. More than 165,000 of these people suffered from respiratory issues, 80,248 from skin issues, and 70,206 from eye discomfort. The PM_2.5_ concentration in February 2023 was 84.7 μg/m^3^, which exceeded the safety standards of both USEPA (35 μg/m^3^) and the Thai (50 μg/m^3^) guidelines, potentially leading to major health consequences (http://air4thai.com/webV3/#/Report, accessed on 20 March 2023).

### 2.2. Data of Future PM_2.5_ Concentration

The data of future PM_2.5_ used in this study was obtained from Amnuaylojaroen et al. [22]. The data were generated using the simulation of the NRCM-Chem modeling system under a climate scenario based on the representative concentration pathway (RCP8.5). The biomass-burning and anthropogenic emission inventories from RCPs were included in the simulation. When compared to the observations, the performance of the PM_2.5_ dataset accurately represents the PM_2.5_ pattern in northern Thailand. The statistical examination of the PM_2.5_ concentrations between the modeled PM_2.5_ dataset and the observation were acceptable, with an index of agreement (0.82), mean-bias (21.9), fractional error (0.043), and residue standard deviation (30.2), and an uncertainty of data with ±6.27 μg/m^3^ [22]. However, the model captures the overestimation of PM_2.5_ predictions. The high bias in PM_2.5_ can impact the prediction of HQ values. Because the average daily dosage (ADD) that is directly related to PM_2.5_ concentration was used to estimate the HQ value, a higher PM_2.5_ concentration would also tend to overpredict the HQ.

### 2.3. Health Risk Assessment

The assessment of health risks is an essential tool for determining the possible negative consequences of pollutant exposure on human health [23,24,25]. This prediction tool examines the measurable data of exposure to assess the risk factor from pollution on human health. The exposure to humans was described by the average daily dosage (ADD), which was calculated as follows in Equation (1):

(1) $$\mathrm{ADD}=\frac{C\;\times \;\mathrm{IR}\;\times \;\mathrm{EF}\;\times \;\mathrm{ED}}{\mathrm{BW}\;\times \;\mathrm{AT}}$$

where IR is the inhalation rate (m^3^/day); C is the pollutant concentration (μg/m^3^); EF is the exposure frequency (days/year); ED is the exposure duration (years); ET is the exposure time (24 h/day); AT is the average exposure time (days); BW is the body weight of the children (kg). The values for these variables were derived from the previous studies, as indicated in Table 1 and Table 2.

Given that individuals in Asia, particularly in Thailand and China, have comparable physical circumstances, the precise values are shown in Table 1. The body weight (BW) and inhalation rate (IR) of children were used by Layton et al. [26] (Table 2), while the average time (AT) included some of the parameters used by Liang et al. [27]. The lifelong exposure of human receptors in children was calculated using an EF of 350 days per year and the hypothesis that the whole population in the research region spends no more than 14 days away from the study area [28,29].

**Table 1:** PM_2.5_ exposure factors used for uptake through the respiratory system.

Factor	Exposure Frequency (EF) (Days/Year)	Exposure Duration (ED) (Years)	Averaging Time (AT) (Days)
Value	350	12	4380
Reference	Morakinyo et al. [28]; Olufemi et al. [29]	Morakinyo et al. [28]; Olufemi et al. [29]	Liang et al. [27]

**Table 2:** Body weight and inhalation in the different age groups of children.

Cohort/Age (Years)	Body Weight (kg)	Inhalation (m^3^/day)
Infant (<1)	7.6	4.5
1–2	13.0	6.8
3–5	18.0	8.3
6–8	26.0	10
Male		
9–11	36.0	14
12–14	50.0	15
15–18	66.0	17
Female		
9–11	36.0	13
12–14	49.0	12
15–18	56.0	12

To determine the non-carcinogenic health risks, the HQ, that is, the ratio of ADD to the reference dose (RfD), was calculated as follows in Equation (2) [11]:

(2) $$\mathrm{Hazard}\;\mathrm{Quotient}\;\left(\mathrm{HQ}\right)=\frac{\mathrm{Average}\;\mathrm{Daily}\;\mathrm{Dose}\;\left(\mathrm{ADD}\right)\left(\frac{\mu g}{\mathrm{kg}}\cdot \mathrm{day}\right)}{\mathrm{Inhalation}\;\mathrm{Reference}\;\mathrm{Dose}\;\left(\mathrm{RfD}\right)\left(\frac{\mu g}{\mathrm{kg}}\cdot \mathrm{day}\right)}$$

The inhalation reference dose (RfD) was calculated as follows in Equation (3):

(3) $$\mathrm{RfD}=\frac{\mathrm{RfC}\;\times \;\mathrm{IR}\;\times \;\mathrm{ET}\;\times \;\mathrm{EF}\;\times \;\mathrm{ED}}{\mathrm{BW}\;\times \;\mathrm{AT}}$$

where the exposure time (ET) = 24 h/day; the inhalation rate (IR) = 0.83 m^3^/h; the exposure frequency (EF) = 350 days/year; the averaging time (AT) = ED ∗ 365 days/year; body weight (BW) = 70 kg; and ED = 30 years. RfC is the safe limit of the inhalation reference proposed by the USEPA National Ambient Air Quality Standard (NAAQS) for PM_2.5_ in 2006, which is 35 μg/m^3^.

The standard for safety is an HQ of 1.0. An HQ less than 1.0 denotes insignificance or “negligible risk”, implying that the pollutant under consideration is unlikely to have unfavorable health consequences, even in a susceptible individual. An HQ greater than 1.0 suggests that there might be certain levels of threat to sensitive persons as a consequence of exposure [11]; however, an HQ greater than 10 implies a substantial chronic risk [30,31].

## 3. Results

### 3.1. Situation of PM_2.5_ Concentrations between 2020 and 2029

**Figure 2:**
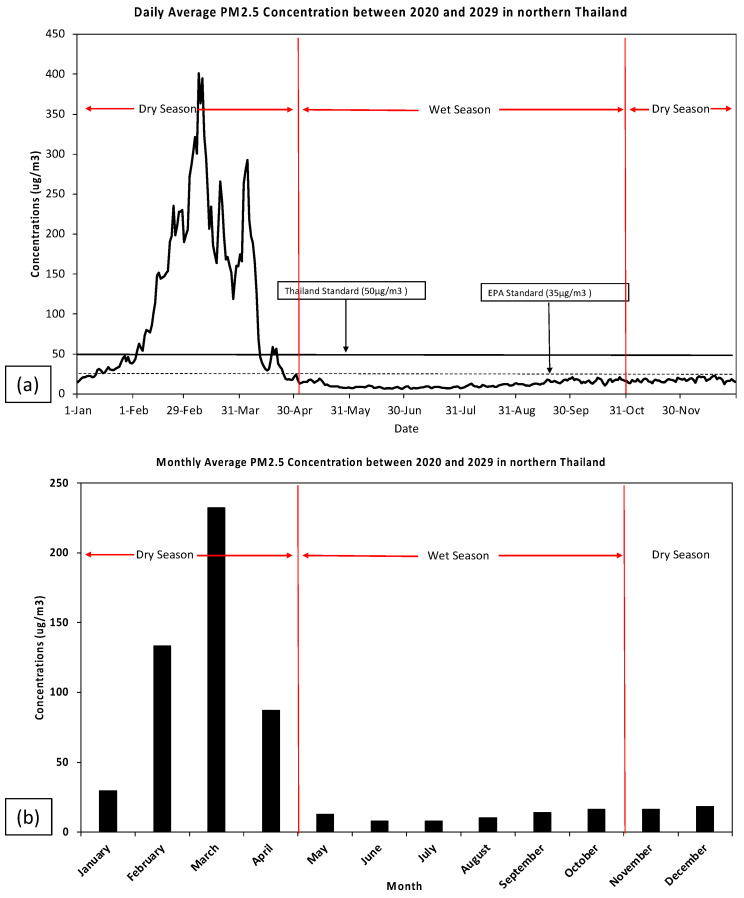
(**a**) Daily and (**b**) monthly means of the PM_2.5_ concentrations in northern Thailand during 2020–2029.

**Figure 3:**
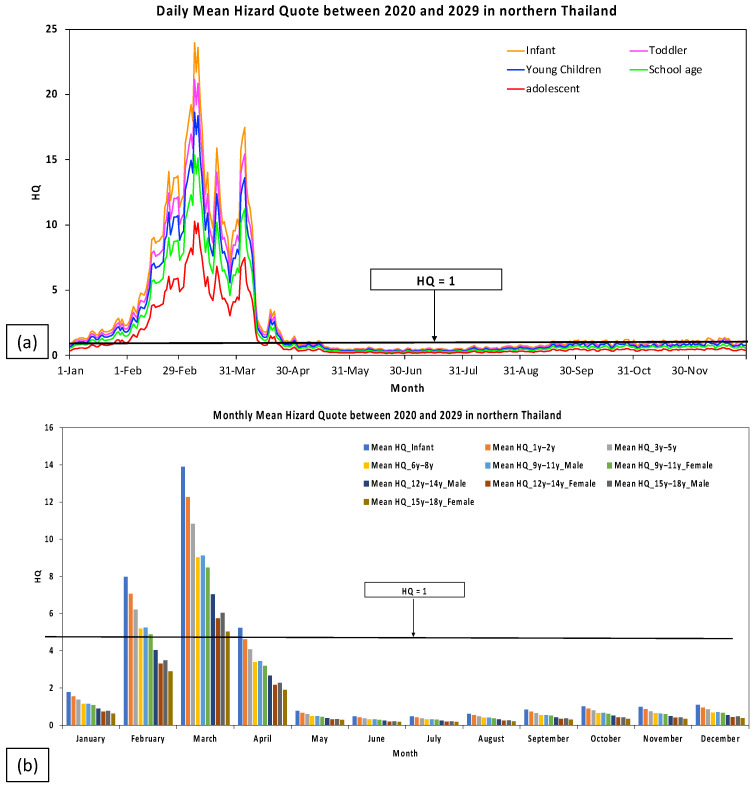
(**a**) Daily means of the HQ related to PM_2.5_ concentrations in the age-related development period and (**b**) the monthly means of the HQ related to PM_2.5_ concentrations in specific groups of ages in children in northern Thailand during 2020–2029 (black line is HQ = 1).

The daily and monthly average between 2020 and 2029 of the PM_2.5_ concentration in northern Thailand is shown in Figure 2 and Figure 3. Between 2020 and 2029, the average daily PM_2.5_ concentrations will greatly surpass both the Thai (50 μg/m^3^) and USEPA (35 μg/m^3^) guidelines, especially during the dry season. The highest daily average PM_2.5_ concentrations were at the beginning of February and ended in April with ranges of 40–400 μg/m^3^ in the future (Figure 2) while the PM_2.5_ concentrations dropped in the rainy season (May to October) and peaked in the dry season (November to December and January to April) (2020–2029) (Figure 3). The worst PM_2.5_ values were detected predominantly between February and March. In northern Thailand, biomass burning emissions govern the seasonal variance of PM_2.5_ [8,22]. PM_2.5_ is extensively distributed by biomass burning including open and waste burning, in advance of the forthcoming rain and rice seeding during the dry season [8].

### 3.2. Hazard Quote in Different Age Groups of Children in Northern Thailand between 2020 and 2029

In this study, the values of HQ were estimated for children in order to evaluate the non-carcinogenic threat of PM_2.5_ in northern Thailand. Figure 3a depicts the daily averages of the HQ associated with PM_2.5_ in northern Thailand throughout the age-related development period of 2020–2029. Militaru and Martinovici [32] classified the age-related development phase. Infants were 1 month to 1 year old, toddlers were 1 to 3 years old, young children were 3 to 6 years old, school-age children were 6 to 12 years old, and adolescents were 12 to 18 years old. During January and April, the daily mean of the HQ was greater than 1. The highest HQ value was discovered in March, suggesting that all age groups of children in northern Thailand face significant danger in the near future. When comparing the age-related development periods, infants had a larger risk than other groups, with an HQ value of 23.96, whereas adolescents had a decreased risk of exposure to PM_2.5_, although the HQ value remained quite high (10.28 for adolescents). Figure 3b depicts the monthly averages of the HQ associated with PM_2.5_ in different age groups of children in northern Thailand between 2020 and 2029. From February through April, the monthly averages of the HQ values were larger than one, suggesting a high risk for all children in northern Thailand. In March, the highest HQ value was discovered in a group of infants. In the dry season, the HQ values between February and April were higher than during November and January. This is likely due to the emissions from the massive biomass burnings from agricultural and waste burning in preparation for the rice planting season. Along with the long-range transport of air pollutants from neighboring countries such as Laos, Vietnam, and Burma, which is induced by meteorological conditions, these factors contribute to the high air pollution in northern Thailand [8].

Table 3 displays the calculated HQ values for PM_2.5_ based on age-related development. HQ values greater than one indicate hazardous exposure situations. Over the entire year, the mean of the HQ was 2.93 ± 1.20, 2.59 ± 1.06, 2.28 ± 0.93, 1.88 ± 0.77, 1.26 ± 0.51 for newborn, toddlers, young children, school age, and adolescents, respectively (Table 3). The averages of the HQ for PM_2.5_ were in the range of 0.48–13.89, 0.42–12.27, 0.37–10.82, 0.31–8.91, and 0.21–5.96 for infants, toddlers, young children, school age, and adolescents, respectively, which were all greater than 1, indicating an intolerable risk to human health. The maximum HQ for infants, toddlers, young children, school age, and adolescents was 0.57–23.96, 0.50–21.16, 0.44–18.66, 0.37–15.37, and 0.24–10.28, respectively. The minimum HQ level for infants, toddlers, young children, school age, and adolescents was 0.38–7.12, 0.34–6.29, 0.30–5.55, 0.24–4.57, and 0.16–3.06, respectively. The HQ values were shown to be greater in February and March than in other months between 2020 and 2029, with March having the highest value (mean HQ = 13.89 for babies, 12.27 for toddlers, 10.82 for young children, 8.91 for school age, and 5.96 for adolescents). Despite the fact that school age and adolescents were at a lower risk than other age groups, the HQ value remained at an undesirable level for human health. Males had greater HQ values than females, as seen by the mean, maximum, and lowest HQ values in Table 4, Table 5 and Table 6. For adolescent males aged 9 to 11 years, 12 to 14 years, and 15 to 18 years, the mean HQ values were 1.92 ± 0.79, 1.48 ± 0.61, and 1.27 ± 0.52, respectively. Females in those age categories had mean HQ values of 1.79 ± 0.73, 1.21 ± 0.50, and 1.06 ± 0.43, respectively (Table 4). For males aged 9 to 11 years, 12 to 14 years, and 15 to 18 years, the minimum HQ values were 0.87 ± 1.54, 0.67 ± 1.19, and 0.58 ± 1.02, respectively. Females in the same age categories had mean HQ values of 0.81 ± 1.43, 0.55 ± 0.97, and 0.48 ± 0.85, respectively (Table 5). The maximum HQ values for males in the age ranges of 9–11 years, 12–14 years, and 15–18 years were 3.66 ± 0.36, 2.82 ± 0.28, and 2.42 ± 0.24, respectively. Females in these age categories had mean HQ values of 3.40 ± 0.33, 2.30 ± 0.23, and 2.02 ± 0.20, respectively (Table 6).

## 4. Discussion

The results of this study indicate that the HQ values of infants were acutely high-risk and higher in comparison to the other groups. This is most likely due to a mix of behavior, environment, and physical factors. They are highly susceptible to the developing fetus and the early years of their lives, when their respiratory systems, organ systems, and brains are still growing. It is uncertain what biological processes induce PM_2.5_ inhalation to cause infant death. Nevertheless, Brook et al. [33] reported that PM_2.5_ exposure likely promotes oxidative stress, systemic inflammation, and blood clotting. As a result, Kannan et al. [34] explained that if a pregnant woman becomes exposed to PM_2.5_, a chain reaction of unfavorable biological reactions may endanger the health of the fetus. Previous studies by Feng et al. [31], and Valentino et al. [35] discovered that PM exposure changed the placental function and structure, potentially impairing fetal development and growth. Simultaneously, Wick et al. [36] revealed that PM_2.5_ was able to pass the cell membrane. As a result, fine particulates that penetrate directly through the placenta would impair the fetus because the immune system of the fetus is still developing [37]. Prenatal exposure to a toxic drug can disrupt the development of many systems necessary for life. For instance, prenatal PM_2.5_ exposure may hinder the development of the cardiovascular and central nervous systems [38]. Furthermore, prenatal PM_2.5_ exposure may disrupt lung maturation by interacting with lung growth, neurogenesis, and differentiation [39]. Prenatal exposure to PM_2.5_ might well be associated with infant mortality, provided that disruptions in the formation and function of biological systems before birth are associated with negative health consequences later in life. Due to the role of exposure time on fetal susceptibility, the magnitude of the adverse effects of PM_2.5_ can fluctuate during the perinatal period [40]. With regard to cognitive function, PM_2.5_ exposure during earlier stages of pregnancy may be associated with severe issues, whereas exposure during the later stages of pregnancy may be associated with inadequacies [38].

Particulate matter is likely to be associated with gender [41]. Due to differences in respiratory symptom rates of growth, disentangling the role of gender in particle pollution connections in children may be more difficult [42]. It has been postulated that observed disparities in poor air quality consequences between males and females were caused by sex-related biological factors such as hormone balancing and body structure or sex differences in behavior patterns, doses, or the accuracy of measurements [43]. Furthermore, because males have a greater death rate than females, the lag in fetal respiratory system development in males might explain their high susceptibility to PM_2.5_ exposure [44,45]. Moreover, long-term prenatal PM_2.5_ exposure disrupts the Ras homolog gene family member A (RhoA) pathway in males [46]. Since the enhanced production of reactive oxygen species is a putative stimulation process of the RhoA system for PM_2.5_, males might be more prone to heart disease when exposed to PM_2.5_ [46,47]. Furthermore, some studies have found that male and female lungs respond differently to air pollution exposure [48,49,50,51]. This likely occurred because male and female lungs differed earlier in fetal and maternal growth all through their lives, with female lungs maturing earlier in terms of surfactant production [52,53]. Women have smaller lungs than men throughout their lives, but their respiratory anatomy is more beneficial, with a larger airway size in comparison to the amount of pulmonary parenchyma. As a result, airway hyperreactivity and asthma are more prevalent in boys than in girls during childhood.

Furthermore, PM_2.5_ does not only have a non-carcinogenic risk on human health; its chemical composition also has an effect on carcinogenic risk on human health. The latest studies demonstrate that the composition of particulate matter is related to human health. For example, Phairuang et al. [54,55] investigated the health risk of PM_0.1_ and its trace elements such as aluminum (Al), barium (Ba), potassium (K), iron (Fe), chromium (Cr), copper (Cu), nickel (Ni), sodium (Na), manganese (Mn), magnesium (Mg), titanium (Ti), lead (Pb), and zinc (Zn) on humans in Bangkok and Hat Yai, Thailand. They discovered that biomass burning was the predominant source of PM and had a high risk for human health in those areas. Additionally, Insian et al. [56] evaluated the respiratory health risk posed by size-fractionated PM-bound polycyclic aromatic hydrocarbons (PAHs) in urban and rural Chiang Mai, northern Thailand. They discovered a rather high respiratory health risk during the smoky haze season in Chiang Mai, Thailand based on toxicity equivalent concentrations of the PAH-bound size-fractionated particulate matters (SPMs) and inhalation cancer risk (ICR). Wang et al. [57] revealed that the health risk assessment of heavy metals showed that non-carcinogenic hazards are not expected to occur, while Cr contributed the highest cancer risk in the industrial areas of China.

## 5. Conclusions

The purpose of this study was to examine health risks among different age groups of children in northern Thailand between 2020 and 2029. The analysis of PM_2.5_ concentrations in the future found that they tended to exceed both the USEPA and Thai guidelines, mainly in the dry season. The air quality in the wet season is expected to be better than the dry season, when PM_2.5_ concentrations tend to be lower. The highest future PM_2.5_ concentrations were detected in ranges of 40 to 400 μg/m^3^ in the dry season, especially in February and March. As a result, the highest concentration of PM_2.5_ was found in March. At the same time, the highest average of HQ values was found with 13.89, 12.27, 10.82, 8.91, and 5.96 for infants, toddlers, young children, school age, and adolescents, respectively, while the HQ of different age groups of children showed the highest value in March with 13.89, 12.27, 10.82, and 9.02 for children aged less than 1 year, 1 to 2 years, 3 to 5 years, and 6 to 8 years, respectively. Meanwhile, the HQ of children aged 9 to 11 years, 12 to 14 years, and 15 to 18 years was in the range of 6.04 to 9.12 for males and 5.03 to 8.47 for females. In conclusion, in general, children of all ages are likely to suffer from PM_2.5_ in the future. Infants are especially at higher risk than other groups of children. Simultaneously, adolescent males tend to be at higher risk than females.

## Figures and Tables

**Table 3:** Mean, maximum, and minimum of the monthly means of the HQ related to PM_2.5_ according to the age-related development period in children during 2020–2029.

Month	Mean					Max					Min				
	Infants	Toddlers	Young Children	School Age	Adolescents	Infants	Toddlers	Young Children	School Age	Adolescents	Infants	Toddlers	Young Children	School Age	Adolescents
January	1.77	1.57	1.38	1.14	0.76	2.83	2.50	2.21	1.82	1.22	0.86	0.76	0.67	0.55	0.37
February	7.98	7.05	6.21	5.12	3.43	14.07	12.43	10.96	9.03	6.04	2.37	2.09	1.84	1.52	1.02
March	13.89	12.27	10.82	8.91	5.96	23.96	21.16	18.66	15.37	10.28	7.12	6.29	5.55	4.57	3.06
April	5.23	4.62	4.07	3.36	2.25	17.48	15.44	13.61	11.21	7.50	1.05	0.93	0.82	0.67	0.45
May	0.77	0.68	0.60	0.49	0.33	1.47	1.29	1.14	0.94	0.63	0.44	0.39	0.34	0.28	0.19
June	0.48	0.42	0.37	0.31	0.21	0.63	0.56	0.49	0.41	0.27	0.38	0.34	0.30	0.24	0.16
July	0.49	0.43	0.38	0.31	0.21	0.57	0.50	0.44	0.37	0.24	0.39	0.34	0.30	0.25	0.17
August	0.62	0.55	0.48	0.40	0.27	0.80	0.70	0.62	0.51	0.34	0.44	0.39	0.34	0.28	0.19
September	0.84	0.74	0.66	0.54	0.36	1.15	1.01	0.89	0.73	0.49	0.58	0.51	0.45	0.37	0.25
October	1.02	0.89	0.81	0.65	0.43	1.20	1.03	1.05	0.79	0.53	0.67	0.67	0.57	0.48	0.32
November	0.99	0.88	0.76	0.64	0.42	1.20	1.10	0.97	0.74	0.49	0.65	0.58	0.54	0.49	0.34
December	1.10	0.95	0.86	0.69	0.47	1.38	1.27	1.12	0.86	0.59	0.71	0.70	0.69	0.53	0.35
Mean	2.93 ± 1.20	2.59 ± 1.06	2.28 ± 0.93	1.88 ± 0.77	1.26 ± 0.51	5.56 ± 2.34	4.92 ± 2.07	4.35 ± 1.82	3.56 ± 1.50	2.39 ± 1.01	1.31 ± 0.55	1.17 ± 0.49	1.03 ± 0.43	0.85 ± 0.35	0.57 ± 0.24

**Table 4:** Mean of the monthly means of the HQ related to PM_2.5_ according to the different age groups of children during 2020–2029.

Month	<1 y	1 y–2 y	3 y–5 y	6 y–8 y	9 y–11 y		12 y–14 y		15 y–18 y	
					Male	Female	Male	Female	Male	Female
January	1.77	1.57	1.38	1.15	1.16	1.08	0.90	0.73	0.77	0.64
February	7.98	7.05	6.21	5.18	5.24	4.87	4.04	3.30	3.47	2.89
March	13.89	12.27	10.82	9.02	9.12	8.47	7.04	5.74	6.04	5.03
April	5.23	4.62	4.07	3.40	3.44	3.19	2.65	2.16	2.28	1.89
May	0.77	0.68	0.60	0.50	0.50	0.47	0.39	0.32	0.33	0.28
June	0.48	0.42	0.37	0.31	0.32	0.29	0.24	0.20	0.21	0.17
July	0.49	0.43	0.38	0.32	0.32	0.30	0.25	0.20	0.21	0.18
August	0.62	0.55	0.48	0.40	0.41	0.38	0.31	0.26	0.27	0.22
September	0.84	0.74	0.66	0.55	0.55	0.51	0.43	0.35	0.37	0.31
October	1.02	0.89	0.81	0.65	0.68	0.62	0.51	0.42	0.43	0.36
November	0.99	0.88	0.76	0.65	0.64	0.60	0.50	0.41	0.43	0.35
December	1.10	0.95	0.86	0.69	0.70	0.66	0.55	0.44	0.48	0.40
Mean	2.93 ± 1.20	2.59 ± 1.06	2.28 ± 0.93	1.90 ± 0.78	1.92 ± 0.79	1.79 ± 0.73	1.48 ± 0.61	1.21 ± 0.50	1.27 ± 0.52	1.06 ± 0.43

**Table 5:** Minimum of the monthly means of the HQ related to PM_2.5_ according to the different age groups of children during 2020–2029.

Month	<1 y	1 y–2 y	3 y–5 y	6 y–8 y	9 y–11 y		12 y–14 y		15 y–18 y	
					Male	Female	Male	Female	Male	Female
January	0.86	0.76	0.67	0.56	0.57	0.53	0.44	0.36	0.38	0.31
February	2.37	2.09	1.84	1.54	1.55	1.44	1.20	0.98	1.03	0.86
March	7.12	6.29	5.55	4.63	4.68	4.34	3.61	2.95	3.10	2.58
April	1.05	0.93	0.82	0.68	0.69	0.64	0.53	0.43	0.46	0.38
May	0.44	0.39	0.34	0.29	0.29	0.27	0.22	0.18	0.19	0.16
June	0.38	0.34	0.30	0.25	0.25	0.23	0.19	0.16	0.17	0.14
July	0.39	0.34	0.30	0.25	0.25	0.24	0.20	0.16	0.17	0.14
August	0.44	0.39	0.34	0.29	0.29	0.27	0.22	0.18	0.19	0.16
September	0.58	0.51	0.45	0.38	0.38	0.35	0.29	0.24	0.25	0.21
October	0.67	0.67	0.57	0.46	0.47	0.46	0.33	0.30	0.33	0.26
November	0.65	0.58	0.54	0.51	0.47	0.47	0.39	0.32	0.30	0.28
December	0.71	0.70	0.69	0.55	0.50	0.47	0.43	0.33	0.36	0.28
Mean	1.31 ± 2.34	1.17 ± 2.07	1.03 ± 1.82	0.86 ± 1.52	0.87 ± 1.54	0.81 ± 1.43	0.67 ± 1.19	0.55 ± 0.97	0.58 ± 1.02	0.48 ± 0.85

**Table 6:** Maximum of the monthly means of the HQ related to PM_2.5_ according to the different age groups of children during 2020–2029.

Month	<1 y	1 y–2 y	3 y–5 y	6 y–8 y	9 y–11 y		12 y–14 y		15 y–18 y	
					Male	Female	Male	Female	Male	Female
January	2.83	2.50	2.21	1.84	1.86	1.73	1.43	1.17	1.23	1.02
February	14.07	12.43	10.96	9.14	9.24	8.58	7.13	5.82	6.12	5.09
March	23.96	21.16	18.66	15.56	15.73	14.61	12.14	9.91	10.42	8.67
April	17.48	15.44	13.61	11.35	11.48	10.66	8.86	7.23	7.60	6.33
May	1.47	1.29	1.14	0.95	0.96	0.89	0.74	0.61	0.64	0.53
June	0.63	0.56	0.49	0.41	0.41	0.39	0.32	0.26	0.27	0.23
July	0.57	0.50	0.44	0.37	0.37	0.35	0.29	0.24	0.25	0.21
August	0.80	0.70	0.62	0.52	0.52	0.49	0.40	0.33	0.35	0.29
September	1.15	1.01	0.89	0.74	0.75	0.70	0.58	0.47	0.50	0.41
October	1.20	1.03	1.05	0.83	0.84	0.76	0.64	0.50	0.56	0.48
November	1.20	1.10	0.97	0.79	0.77	0.78	0.61	0.49	0.51	0.44
December	1.38	1.27	1.12	0.86	0.90	0.82	0.75	0.56	0.64	0.50
Mean	5.56 ± 0.55	4.92 ± 0.49	4.35 ± 0.43	3.61 ± 0.36	3.66 ± 0.36	3.40 ± 0.33	2.82 ± 0.28	2.30 ± 0.23	2.42 ± 0.24	2.02 ± 0.20

## Data Availability

All data generated or analyzed during this study are included in this published article.
